# Characterization of Peptides Found in Unprocessed and Extruded Amaranth (*Amaranthus hypochondriacus*) Pepsin/Pancreatin Hydrolysates

**DOI:** 10.3390/ijms16048536

**Published:** 2015-04-16

**Authors:** Alvaro Montoya-Rodríguez, Jorge Milán-Carrillo, Cuauhtémoc Reyes-Moreno, Elvira González de Mejía

**Affiliations:** 1 North West Regional Program in Biotechnology, University of Sinaloa, FCQB-UAS, AP 1354, CP 80000 Culiacán, Sinaloa, Mexico; E-Mails: amontoyar5@gmail.com (A.M.-R.); jmilanc@gmail.com (J.M.-C.); creyes@uas.edu.mx (C.R.-M.); 2 Food Science and Human Nutrition, University of Illinois at Urbana-Champaign, 228 ERML, MC-051, 1201 West Gregory Drive, Urbana, IL 61801, USA

**Keywords:** amaranth, hydrolysis, bioactive peptides, extrusion

## Abstract

The objectives of this study were to characterize peptides found in unprocessed amaranth hydrolysates (UAH) and extruded amaranth hydrolysates (EAH) and to determine the effect of the hydrolysis time on the profile of peptides produced. Amaranth grain was extruded in a single screw extruder at 125 °C of extrusion temperature and 130 rpm of screw speed. Unprocessed and extruded amaranth flour were hydrolyzed with pepsin/pancreatin enzymes following a kinetic at 10, 25, 60, 90, 120 and 180 min for each enzyme. After 180 min of pepsin hydrolysis, aliquots were taken at each time during pancreatin hydrolysis to characterize the hydrolysates by MALDI-TOF/MS-MS. Molecular masses (MM) (527, 567, 802, 984, 1295, 1545, 2034 and 2064 Da) of peptides appeared consistently during hydrolysis, showing high intensity at 10 min (2064 Da), 120 min (802 Da) and 180 min (567 Da) in UAH. EAH showed high intensity at 10 min (2034 Da) and 120 min (984, 1295 and 1545 Da). Extrusion produced more peptides with MM lower than 1000 Da immediately after 10 min of hydrolysis. Hydrolysis time impacted on the peptide profile, as longer the time lower the MM in both amaranth hydrolysates. Sequences obtained were analyzed for their biological activity at BIOPEP, showing important inhibitory activities related to chronic diseases. These peptides could be used as a food ingredient/supplement in a healthy diet to prevent the risk to develop chronic diseases.

## 1. Introduction

Amaranth (*Amaranthus hypochondriacus*) is a native grain of Mexico, where it has been used since pre-Columbian civilizations by Aztecs and Mayas [1,2,3]. The importance of amaranth has resurged in the past 20 years because of its agricultural features, since it is a fast growing cultivar with tolerance to drought conditions. In addition, amaranth can grow in poor soils where common crops cannot grow. Likewise, amaranth has important nutritional and nutraceutical properties [4,5,6]. Amaranth grain has excellent chemical composition with high concentration of proteins (13%–19%), which is higher than in cereals [7,8,9,10]. Globulins, albumins and glutelins are the principal proteins in amaranth [11]. These proteins have great nutritional quality and value, with excellent balance of essential amino acids such as lysine and sulfur-containing amino acids, which are deficient in traditional cereals and legumes, respectively [12,13,14]. The quality of amaranth proteins is comparable to the optimum protein reference pattern in the human diet, almost reaching the requirements according to FAO/WHO [15,16]. It also presents high digestibility (90% of the proteins are digested) [17]. These characteristics suggest that amaranth could be used in a mixture or combination with cereals to improve the quality of the protein and have a better nutritive food [18]. The combination of amaranth and maize flour in a ratio 50:50 almost reaches the 100 score on the nutritional scale. The limiting amino acids in amaranth seed are leucine, isoleucine and valine. However, they are not a serious problem, since they are in excess in most common grains [12]. In addition, amaranth does not contain gluten which is suitable for people with celiac disease [19]. Due to the high protein concentration, amaranth could be a source of bioactive peptides. Bioactive peptides are inactive within the parent protein; however, with enzymatic digestion or food processing, peptides can act as physiological modulators of metabolism releasing these bioactive peptides [20]. Extrusion is a high temperature-short-time food processing technology that has been used to get precooked flours with high nutritional value [21] and also it shows a positive effect on antioxidant capacity [22]. Some studies of amaranth have reported the presence of peptides with biological activities such as anti-hypertensive, anti-oxidative, anti-thrombotic, among others [11,23,24]. Our group has evaluated the biological activity of the peptides present in the hydrolysates from unprocessed amaranth (UAH) and extruded amaranth (EAH) [25,26]. We found that peptides present on these hydrolysates have anti-inflammatory and anti-atherosclerotic potential. However, the specific characteristics of these peptides were not reported.

Hence, the objectives of this study were to perform an extensive characterization of peptides found in UAH and EAH, and to determine the effect of the hydrolysis time on the profile of the peptides produced. For the first time, this manuscript provides novel information on the time-course of the effect of gastrointestinal enzymes to produce peptides from amaranth proteins with potential biological activities throughout hydrolysis. Peptides were characterized by mass spectrometry and their presence in amaranth proteins was identified. This complete characterization of peptides during simulated gastrointestinal digestion of amaranth proteins is unique since it has not been provided in previous publications.

## 2. Results and Discussion

### 2.1. Pepsin and Pancreatin Hydrolysates

Unprocessed and extruded amaranth flour hydrolysates were obtained after 360 min of hydrolysis; 180 min with pepsin and 180 min with pancreatin. After 360 min of hydrolysis, the freeze dried material was obtained and the mass yield (weight of the hydrolysate in grams/weight of original material in grams) was 31% *w*/*w*.

The protein electrophoretic profile of UAH gave a distinct protein pattern, characteristic of amaranth proteins [27]. For instance, the 28 kDa band corresponded to amaranth albumins that are water soluble proteins, while 7S and 11S globulin fractions corresponded to proteins of molecular masses 33 and 57 kDa, respectively. The extrusion process changed the protein profile of the amaranth flour showing barely detectable amaranth albumins and 7S and 11S globulins. Extrusion also led to the production of smaller molecular mass proteins and peptides. As expected, the degree of hydrolysis increased in relation to digestion time. Maximum degree of hydrolysis was reached after pancreatin digestion at 360 min for both flours, UAH and EAH. After 180 min of pepsin hydrolysis, most large molecular mass proteins were converted to smaller proteins of molecular mass less than 37 kDa, while pancreatin digestion led to transformation of large molecular mass proteins leading to the production of peptides with molecular mass less than 10 kDa [28].

### 2.2. MALDI-TOF Hydrolysates Characterization

The unprocessed amaranth hydrolysates characterized by MALDI-TOF are shown in Table 1 and Table 2. Table 1 shows the hydrolysates 10, 25 and 60 min after hydrolysis. The MM of these peptides was between 520 and 3580 Da for these three hydrolysate times. More peptides were present after 10 and 60 min of hydrolysis than after 25 min of hydrolysis. However, the hydrolysate at 25 and 60 min showed peptides with lower MM than the hydrolysate at 10 min. Table 2 shows the hydrolysates at 90, 120 and 180 min after pancreatin hydrolysis. Peptides from 511 to 3900 Da appeared at 90, 120 and 180 min. However, peptides longer than 1200 Da were more abundant only at 120 min.

Table 3 and Table 4 show the extruded amaranth hydrolysates characterized by MALDI-TOF. Table 3 shows the hydrolysates at 10, 25 and 60 min after pancreatin hydrolysis. Peptides with MM between 511 and 4100 Da were present at these hydrolysis times. However, peptides longer than 2100 Da appear with a weak intensity. Table 4 shows the hydrolysates at 90, 120 and 180 min after pancreatin hydrolysis. Peptides from 511 to 3800 Da appear at 90, 120 and 180 min. However, peptides longer than 2100 Da appear with weak intensity and were only present at 120 min.

The hydrolysis time impacted on the MM of the peptides in both flours (Table 5). More peptides with low MM appeared after 90 min of hydrolysis in both flours. However, in the extruded amaranth hydrolysates, peptides with low MM appeared immediately after 10 min of hydrolysis. This could be explained by the extrusion process since higher levels of free amino acids were present and the fact that during protein enzymatic hydrolysis amino acids were released [28,29]; extrusion could cause protein hydrolysis, producing small peptides during the process. In addition, the extrusion process produced partial denaturation of the proteins, being the proteins more available for the enzyme action. The extrusion process showed more peptides with lower MM at the end of the hydrolysis and also more peptides with biologically active sequences.

MM of 527, 567, 802, 984, 1295, 1545, 2034 and 2064 Da appeared consistently in the whole hydrolysis time in both flours. MALDI-TOF analysis showed high intensity peaks of MM at 10 min (2064 Da), 120 min (802 Da) and 180 min (567 Da) in UAH, while EAH showed high intensity at 10 min (2034 Da) and 120 min (984, 1295 and 1545 Da). In addition, most of these peptides showed a high percent of area under the curve in the MALDI-TOF spectrum analysis. Due to the fact that these peptides appeared consistently throughout hydrolysis, the time where each one showed the highest intensity was selected to be analyzed by MS-MS to know their sequence.

### 2.3. Characterization of UAH and EAH Amaranth Peptides by MS-MS

**Figure 1:**
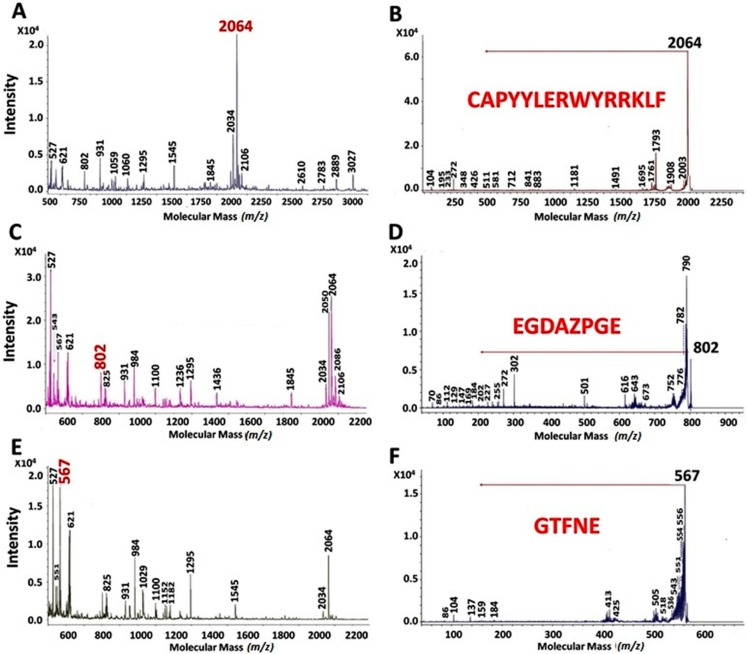
MALDI-TOF analyses of unprocessed amaranth hydrolysates after 10 min (**A**); 120 min (**C**) and 180 min (**E**) with pancreatin. The peaks with molecular mass of 2064 (**B**); 802 (**D**) and 567 (**F**) were analyzed by MS-MS. These peaks were present along the hydrolysis times and showed the highest intensity at these hydrolysis times.

After MALDI-TOF analysis, peaks with the highest intensity were selected to be fragmented by MS-MS to know their peptide sequence. Figure 1 shows the peaks with the highest intensity found in UAH. Figure 1A shows the MALDI-TOF spectrum for the peptide with MM of 2064 Da and Figure 1B shows the MS-MS spectrum for the same peptide. After the MS-MS fragmentation, analysis of the spectrum shows that the potential sequence of the peptide was CAPYYLERWYRRKLF (2064 Da). Likewise, Figure 1C shows the MALDI-TOF spectrum for the peptide with MM of 802 Da and the MS-MS fragmentation was shown in Figure 1D, with a sequence for the peptide EGDAZPGE (802 Da). Figure 1E shows the MALDI-TOF spectrum for the peptide with MM of 567 Da and in Figure 1F the MS-MS spectrum of the peptide sequence with MM of 567 Da, GTFNE.

**Table 1:** Characterization by MALDI-TOF of peptides from unprocessed amaranth pancreatin hydrolysates at 10, 25 and 60 min.

MM (Da)	10 RA			25 RA			60 RA		
	Intensity	Area	% of Area	Intensity	Area	% of Area	Intensity	Area	% of Area
520	-	-	-	8533	2520	3.47	-	-	-
522	2796	698	0.97	16,061	5044	6.95	-	-	-
527	3847	938	1.31	5396	1374	1.89	23,053	4712	5.65
543	-	-	-	-	-	-	4617	1019	1.22
563	-	-	-	6290	1774	2.44	6816	1433	1.72
567	2805	826	1.15	11,598	3200	4.41	13,107	2797	3.35
571	-	-	-	3607	1051	1.45	3881	795	0.95
613	-	-	-	3298	1000	1.38	3515	774	0.93
616	-	-	-	4222	1357	1.87	4897	1117	1.34
617	-	-	-	10,310	3256	4.48	12,614	3045	3.65
619	2871	920	1.28	3505	1132	1.56	4704	1078	1.29
620	-	-	-	3905	1392	1.92	5409	1577	1.89
621	2911	911	1.27	10,788	3413	4.70	13,871	3477	4.17
623	-	-	-	-	-	-	3763	1152	1.38
625	-	-	-	4098	1435	1.98	5854	1470	1.76
802	2585	1005	1.40	3490	1541	2.12	3108	971	1.16
821	-	-	-	-	-	-	2560	771	0.92
825	-	-	-	3957	1729	2.38	5008	1591	1.91
829	-	-	-	3118	1561	2.15	4294	1408	1.69
931	4251	1907	2.66	4511	2259	3.11	3073	1065	1.28
984	-	-	-	-	-	-	2670	1094	1.31
1028	1892	754	1.05	-	-	-	-	-	-
1045	1405	720	1.00	-	-	-	-	-	-
1060	1952	1057	1.47	3020	1803	2.48	-	-	-
1160	1994	1101	1.53	1850	1217	1.68	-	-	-
1234	-	-	-	-	-	-	2487	1424	1.71
1295	2130	1393	1.94	3851	2850	3.93	3517	2107	2.52
1435	-	-	-	-	-	-	1668	1292	1.55
1545	3343	2703	3.77	2808	2565	3.53	1433	1134	1.36
1845	990	2515	3.51	-	-	-	1369	3123	3.74
1899	-	-	-	1124	1503	2.07	-	-	-
2014	2316	2825	3.94	912	1166	1.61	821	1040	1.25
2034	6795	8809	12.28	3669	4628	6.37	4647	5384	6.45
2050	1260	2559	3.57	-	-	-	1292	2289	2.74
2056	-	-	-	-	-	-	803	1358	1.63
*2064*	*22,157*	*25,398*	*35.40*	9809	13,092	18.03	16,883	20,859	24.99
2071	-	-	-	-	-	-	3278	3854	4.62
2076	3048	4023	5.61	2397	3203	4.41	-	-	-
2086	1684	2013	2.81	-	-	-	2818	3384	4.05
2106	1790	2404	3.35	1356	1873	2.58	1888	2063	2.47
2609	577	971	1.35	-	-	-	-	-	-
2783	491	732	1.02	-	-	-	-	-	-
2889	1142	1952	2.72	582	1299	1.79	-	-	-
3027	1380	2612	3.64	967	2367	3.26	331	656	0.79
3267	-	-	-	-	-	-	395	785	0.94
3581	-	-	-	-	-	-	583	1369	1.64

MM = Molecular mass; Da **=** Daltons; RA **=** Unprocessed flour with pancreatin; 10, 25, 60 **=** Minutes of hydrolysis with pancreatin after 180 with pepsin. Italics indicate the mass of the peptides with the highest intensity or % area.

**Table 2:** Characterization by MALDI-TOF of peptides from unprocessed amaranth pancreatin hydrolysates at 90, 120 and 180 min.

MM (Da)	90 RA			120 RA			180 RA		
	Intensity	Area	% of Area	Intensity	Area	% of Area	Intensity	Area	% of Area
511	-	-	-	4456	1147	0.97	-	-	-
518	3942	911	1.86	-	-	-	-	-	-
520	10,311	2162	4.42	7918	1787	1.51	-	-	-
522	16,113	3407	6.97	12,115	2917	2.47	-	-	-
527	28,308	5819	11.90	30,901	6863	5.81	17,708	4186	7.01
543	6887	2151	4.40	6785	1717	1.45	3578	952	1.59
544	3402	765	1.57	-	-	-	-	-	-
551	3483	1701	3.48	-	-	-	3139	1601	2.68
563	8122	1639	3.35	6055	1348	1.14	6418	1641	2.75
*567*	15,285	3149	6.44	1290	2661	2.25	*19,127*	*2849*	*4.77*
571	4652	888	1.82	3815	762	0.65	3438	875	1.47
605	3702	855	1.75	-	-	-	2429	689	1.15
613	4086	923	1.89	-	-	-	3113	950	1.59
614	3559	942	1.93	-	-	-	-	-	-
616	5833	1374	2.81	3936	1192	1.01	4275	1254	2.10
617	14,666	3479	7.12	9341	2555	2.16	9820	2750	4.61
619	5729	1301	2.66	4213	1279	1.08	3311	1055	1.77
620	6434	1842	3.77	12,159	3405	2.88	3695	1151	1.93
621	16,898	4122	8.43	-	-	-	10,019	3052	5.11
623	4467	1394	2.85	-	-	-	2696	856	1.43
625	6945	1724	3.53	4569	1456	1.23	3693	1298	2.17
642	5163	1196	2.45	-	-	-	-	-	-
*802*	-	-	-	*8474*	*3029*	*2.57*	3646	1191	2.00
821	3265	957	1.96	-	-	-	-	-	-
825	6533	1986	4.06	4843	1616	1.37	3710	1403	2.35
829	5582	1748	3.58	4178	1447	1.23	2822	1227	2.06
931	-	-	-	5546	2509	2.12	2293	871	1.46
984	-	-	-	8651	4514	3.82	7974	3494	5.85
1028	-	-	-	-	-	-	3241	1271	2.13
1029	-	-	-	-	-	-	2988	1344	2.25
1100	-	-	-	3852	2177	1.84	1971	991	1.66
1103	-	-	-	-	-	-	1592	732	1.23
1152	-	-	-	-	-	-	1622	870	1.46
1161	-	-	-	-	-	-	1674	918	1.54
1182	-	-	-	-	-	-	1802	1041	1.74
1236	2061	1168	2.39	4094	2155	1.83	-	-	-
1295	2045	1276	2.61	5722	3977	3.37	5641	3503	5.87
1436	-	-	-	2914	2160	1.83	-	-	-
1545	-	-	-	-	-	-	1916	1568	2.63
1845	-	-	-	2880	6193	5.24	-	-	-
2034	1638	1764	3.61	4791	5546	4.70	849	1040	1.74
2046	-	-	-	1064	1693	1.43	-	-	-
2050	-	-	-	2476	4615	3.91	-	-	-
2056	-	-	-	1428	2673	2.26	-	-	-
*2064*	5467	6052	12.38	17,250	25,782	21.84	7614	9419	15.78
2076	1412	1592	3.26	2644	3473	2.94	-	-	-
2086	1374	1414	2.89	6138	7024	5.95	-	-	-
2102	-	-	-	1306	1510	1.28	-	-	-
2106	838	944	1.93	2060	2389	2.02	-	-	-
2118	-	-	-	1463	1556	1.32	-	-	-
3267	936	1670	3.42	877	1697	1.44	873	1812	3.04
3585	-	-	-	-	-	-	154	411	0.69
3910	-	-	-	278	598	0.51	143	377	0.63
3931	-	-	-	302	653	0.55	-	-	-

MM = Molecular mass; Da = Daltons; RA = Unprocessed flour with pancreatin; 90, 120, 180 = Minutes of hydrolysis with pancreatin after 180 with pepsin. Italics indicate the mass of the peptide with the highest intensity or % area.

**Table 3:** Characterization by MALDI-TOF of peptides from extruded amaranth pancreatin hydrolysates at 10, 25 and 60 min.

MM (Da)	10 EA			25 EA			60 EA		
	Intensity	Area	% of Area	Intensity	Area	% of Area	Intensity	Area	% of area
511	4023	1200	1.18	-	-	-	-	-	-
*527*	*44,864*	*8348*	*8.18*	32,294	7546	5.70	32,765	7195	8.73
543	9619	2363	2.32	8061	1853	1.40	7333	1656	2.01
563	5331	1303	1.28	10,371	2528	1.91	6213	1356	1.64
567	8233	2054	2.01	10,797	4652	3.51	12,111	2745	3.33
571	2406	646	0.63	6062	1416	1.07	4350	949	1.15
613	2705	638	0.63	5618	1366	1.03	3196	841	1.02
616	3477	853	0.84	7149	1823	1.38	4765	1241	1.51
617	8638	2247	2.20	17,484	4759	3.60	10,565	2863	3.47
619	2880	908	0.89	6468	1833	1.38	3719	1172	1.42
620	4233	1065	1.04	7118	2387	1.80	4597	1078	1.31
621	9408	2591	2.54	19,361	5429	4.10	12,254	3405	4.13
623	2537	815	0.80	5323	1707	1.29	3638	1120	1.36
625	3861	1102	1.08	8395	2545	1.92	5284	1613	1.96
802	-	-	-	-	-	-	6918	3091	3.75
821	-	-	-	4037	1325	1.00	-	-	-
825	3562	1289	1.26	7130	2505	1.89	5265	1793	2.17
829	2853	1068	1.05	5784	2196	1.66	4384	1658	2.01
931	-	-	-	-	-	-	4247	2236	2.71
984	-	-	-	-	-	-	3431	1938	2.35
1029	1751	1067	1.05	-	-	-	-	-	-
1058	-	-	-	2853	2328	1.76	-	-	-
1152	-	-	-	-	-	-	2161	1564	1.90
1175	-	-	-	-	-	-	2287	1244	1.51
1295	-	-	-	2737	2253	1.70	3010	2317	2.81
1510	-	-	-	-	-	-	-	1719	2.08
1545	-	-	-	6383	6705	5.07	1520	3837	4.65
2014	-	-	-	1036	1568	1.18	3760	6488	7.87
*2034*	1618	2496	2.45	*6796*	*9606*	*7.26*	4402	14,649	17.77
2064	4010	6399	6.27	15,951	25,295	19.11	10,248	3654	4.43
2076	1055	1764	1.73	3864	5300	4.00	2681	2179	2.64
2106	-	-	-	2137	2953	2.23	1555	3359	4.07
3267	-	-	-	306	604	0.46	1184	1749	2.12
3581	-	-	-	561	1220	0.92	555	1749	2.12
4046	1119	13,322	13.06	490	6246	4.72	-	-	-
4059	813	11,555	11.33	292	3704	2.80	-	-	-
4080	1706	22,887	22.44	361	5643	4.26	-	-	-
4098	-	-	-	280	4109	3.10	-	-	-
4105	963	14033	13.76	-	-	-	-	-	-
4121	-	-	-	569	8944	6.76	-	-	-

MM = Molecular mass; Da = Daltons; EA = Extruded flour with pancreatin; 10, 25, 60 = Minutes of hydrolysis with pancreatin after 180 min with pepsin. Italics indicate the mass of the peptide with the highest intensity or % area.

**Table 4:** Characterization by MALDI-TOF of peptides from extruded amaranth pancreatin hydrolysates at 90, 120 and 180 min.

MM (Da)	90 EA			120 EA			180 EA		
	Intensity	Area	% of Area	Intensity	Area	% of Area	Intensity	Area	% of Area
511	3431	1035	1.28	4047	1672	1.44	2889	1225	2.14
527	33,333	6872	8.49	35,283	11664	10.07	27,734	6793	11.85
543	10,127	2156	2.67	10,824	2798	2.41	5629	1466	2.56
551	4242	1312	1.62	-	-	-	-	-	-
563	6457	1394	1.72	6494	1893	1.63	4824	1357	2.37
567	11,131	2447	3.02	11,454	3150	2.72	8491	2227	3.89
571	3204	634	0.78	3222	973	0.84	-	-	-
613	3580	854	1.06	3147	893	0.77	2492	731	1.28
616	4082	1250	1.55	4563	1156	1.00	2923	926	1.62
617	12,157	3044	3.76	9682	2752	2.37	8856	2501	4.36
619	4303	1077	1.33	3670	995	0.86	2839	920	1.61
620	5250	1438	1.78	4300	1373	1.18	3295	998	1.74
621	13,401	3485	4.31	10,859	3188	2.75	8726	2567	4.48
623	3494	1106	1.37	3165	1001	0.86	2480	784	1.37
625	5315	1439	1.78	4293	1402	1.21	3410	1121	1.96
663	-	-	-	3324	1116	0.96	-	-	-
802	7650	3318	4.10	6860	2890	2.49	7342	3409	5.95
825	4532	1523	1.88	4283	1651	1.42	2999	1248	2.18
829	4248	1491	1.84	3481	1514	1.31	2313	1077	1.88
931	5171	2313	2.86	3450	1701	1.47	3715	1977	3.45
*984*	6532	2978	3.68	*9331*	*4683*	*4.04*	9271	5183	9.04
1013	-	-	-	2806	1686	1.45	-	-	-
1030	-	-	-	-	-	-	1912	1081	1.89
1058	-	-	-	2526	1653	1.43	-	-	-
1100	-	-	-	3179	1841	1.59	3055	1820	3.18
1152	2864	1717	2.12	4156	2607	2.25	2741	1879	3.28
1175	2087	1039	1.28	2523	1638	1.41	-	-	-
1182	2422	1394	1.72	4423	2898	2.50	3769	2617	4.57
*1295*	3403	2304	2.85	*7890*	*5901*	*5.09*	3493	3048	5.32
1510	1718	1489	1.84	1582	1617	1.40	-	-	-
*1545*	3304	2987	3.69	*10,228*	*10,310*	*8.90*	2597	3021	5.27
1716	1209	1435	1.77	-	-	-	-	-	-
2034	3308	4371	5.40	2113	3570	22.03	-	-	-
2064	8506	12,656	15.64	14,575	25,523	2.33	3261	5052	8.81
2076	4310	6202	7.67	1898	2703	1.69	-	-	-
2106	2700	3706	4.58	1266	1964	1.88	-	-	-
3267	206	434	0.54	882	2181	0.68	859	2286	3.99
3581	-	-	-	284	785	0.46	-	-	-
3799	-	-	-	167	536	100	-	-	-

MM = Molecular mass; **DA** = Daltons; EA = Extruded flour with pancreatin; 90, 120, 180 = Minutes of hydrolysis with pancreatin after 180 min with pepsin. Italics indicate the mass of the peptides with the highest intensity or % area.

**Table 5:** Effect of hydrolysis time and extrusion process on peptide profile of amaranth proteins. Higher occurrence of peptides with low molecular mass appears after 90 min of hydrolysis.

Hydrolysis Time (min)												
-	UNPROCESSED (%AUC)						EXTRUDED (%AUC)					
MM (Da)	10	25	60	90	120	180	10	25	60	90	120	180
0–1000	10.04	48.26	37.55	72.75	35.74	57.63	27.93	34.66	46.02	50.89	41.82	63.70
1000–2000	14.28	13.69	10.88	3.86	14.11	20.50	1.05	8.53	12.95	15.28	26.02	23.50
2000–3000	72.04	34.79	48.20	18.43	47.65	17.52	10.45	33.79	36.78	33.29	29.13	8.81
>3000	3.64	3.26	3.37	4.99	2.50	4.36	60.58	23.02	4.22	0.54	3.02	3.99

MM: Molecular mass; Da: Daltons; AUC: Area under curve; min: Minutes.

**Figure 2:**
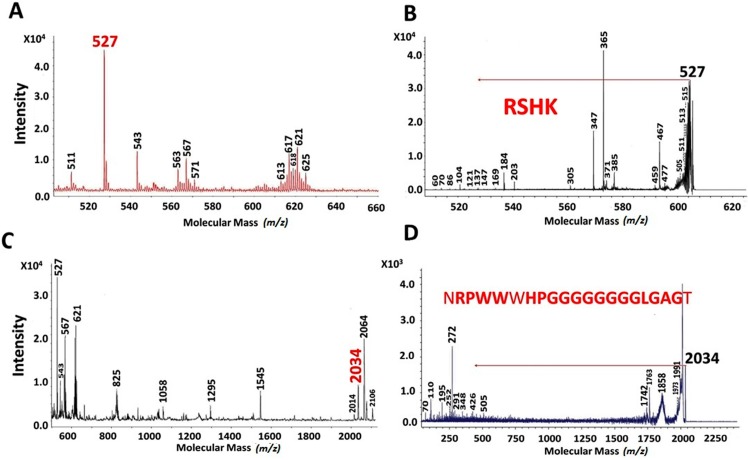
MALDI-TOF analyses of extruded amaranth hydrolysates after 10 min (**A**) and 25 min (**C**) with pancreatin. The peaks with molecular mass of 527 (**B**) and 2034 (**D**) were analyzed by MS-MS. These peaks were present along the hydrolysis times and showed the highest intensity at these hydrolysis times.

**Figure 3:**
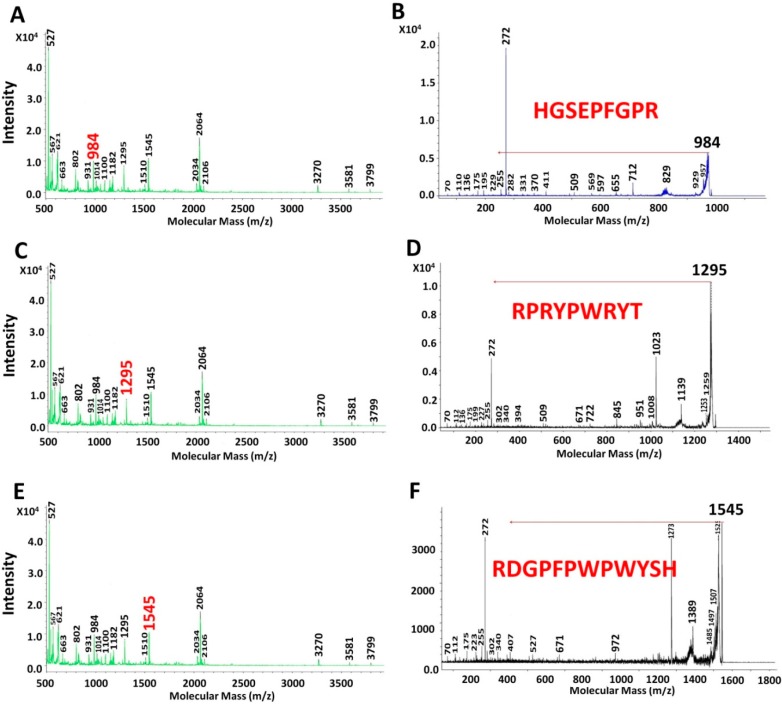
MALDI-TOF analyses of extruded amaranth hydrolysates after 120 min (**A**,**C**,**E**) with pancreatin. The peaks with molecular mass of 984 (**B**); 1295 (**D**); and 1545 (**F**) were analyzed by MS-MS. These peaks were present along the hydrolysis and showed the highest intensity at these hydrolysis times.

The peaks with highest intensity present in EAH are shown in Figure 2 and Figure 3. Figure 2A,C show the MALDI-TOF spectrum for the peptides with MM of 527 and 2034 Da, respectively. While in Figure 2B,D their MS-MS spectra are shown, respectively. The sequence for these MM were RSHK (527 Da) and NRPWWWHPGGGGGGGGLGAGT (2034 Da). Figure 3 shows the MALDI-TOF and MS-MS spectrum of 984 Da (3A and 3B, respectively), 1295 Da (3C and 3D, respectively) and 1545 Da (3E and 3F, respectively). The sequences of these 3 peptides were HGSEPFGPR, RPRYPWRYT and RDGPFPWPWYSH, respectively. All of these peptides, found in both flours, unprocessed and extruded, are hydrophobic and most of them (except EGDAZPGE and GTFNE) present positive net charge [25]. 

It is important to mention that, based on the MS-MS identification of some of the peptide sequences, amino acid sequences present in proteins such as 11S globulin seed storage protein, polyamine oxidase, glucosyltransferase, among others, were found.

Silva-Sánchez *et al.* [11] and Velarde-Salcedo *et al.* [30] also found peptides derived from the hydrolysis of amaranth (*Amaranthus hypochondriacus* L.) proteins by MS-MS analysis.

Peptide sequences in amaranth have been associated with potential biological activities such as antioxidant capacity; inhibitors of dipeptidyl peptidase IV (DPP-IV) important in the management of diabetes; angiotensin converting enzyme inhibitor; and antithrombotic activity [31]. Montoya-Rodriguez *et al.* [25,26] reported that extruded amaranth hydrolysates showed potential anti-inflammatory and anti-atherosclerotic activity *in vitro*, probably attributed to the production of bioactive peptides during the extrusion process. The degree of hydrolysis had a differential effect on the antioxidant capacity of UAH and EAH [25,26]. For instance, in UAH maximum antioxidant capacity was reached after 120 min of pepsin digestion while maximum antioxidant capacity was reached after 25 min of pancreatin digestion. This can be attributed to different antioxidant characteristics of the peptides generated during hydrolysis; different peptides have different antioxidant capacity dictated by their amino acid composition. For instance, the most reactive amino acids are sulfur-containing Met and Cys, aromatic Trp, Phe, and His, attributed to its imidazole ring. Most of the molecular masses of the peptides ranged from 500 to 1800 kDa showed high values of antioxidant capacity. Peptides with similar molecular masses were found in this study. Aside from the degree of hydrolysis, extrusion affected the antioxidant capacity of the hydrolysates. Pepsin hydrolysates from UAH had higher antioxidant capacity than hydrolysates from EAH. Extrusion can also lead to interactions between proteins and other macromolecules such as starch and lipids. Amaranth grain has a high starch content (60%–70%); which makes the protein less available as an antioxidant. However, when pancreatin was used, those interactions could have been hydrolyzed freeing the generated peptides hence the observed increase in antioxidant capacity after pancreatin hydrolysis in EAH; pancreatin is a mixture of enzymes including amylases, lipases and proteases, leading to almost complete hydrolysis and production of small molecules. More research is needed to determine the structural changes of amaranth protein-carbohydrates interactions under the extrusion conditions used in this study.

Tovar-Pérez *et al.* [32] reported peptides from amaranth hydrolysates with ACE-inhibitory activity. Vecchi and Añon [33] reported that peptides present in the globulin 11S of the amaranth showed ACE-inhibitory activity. Maldonado-Cervantes *et al.* [34] reported that the amaranth lunasin-like peptide, obtained from fragmented amaranth proteins, showed potential anti-cancer activity. Enzymatic hydrolysis or food processing could be used to obtain peptides with potential biological activity; however, the method used will depend on several factors such as the bioactive peptide of interest to be released and intended use of the peptide. Although the complete mechanism of absorption and the bioavailability of specific peptides are still under investigation, there is enough evidence to conclude that food bioactive peptides are bioavailable and can be absorbed into the body [35].

The significance and importance of this study was that it provides new information on the kinetics of hydrolysis of amaranth proteins, exposed to gastrointestinal enzymes, to produce peptide sequences with potential biological activities.

## 3. Experimental Section

### 3.1. Materials

The amaranth (*Amaranthus hypochondriacus*) grain was purchased at a local market in Temoac, Morelos, Mexico. The amaranth grains were packed in lots of 1 kg and storage in refrigeration (5–10 °C) until their use.

### 3.2. Extrusion Process

The extruded amaranth flours were obtained according to procedure recommended by Milán-Carrillo *et al.* [22] The amaranth grains (1 kg lots) were milled and mixed with lime (0.21 g Ca(OH)_2_/100 g amaranth) and conditioned with purified water to reach a moisture content of 28%. Each lot was packed in a polyethylene bag and stored at 4 °C for 8 h. Before extrusion, the flours were tempered at 25 °C for 4 h. A single screw laboratory extruder Model 20 DN (CW Brabender Instruments, Inc., South Hackensack, NJ, USA) with a 19 mm screw-diameter; length to diameter 20:1; nominal compression ratio 2:1; and die opening of 3 mm was used. The inner barrel was grooved to ensure zero slip at the wall. The temperature in the barrel was the same for the three zones and the end zone was cooled by air. A third zone, at the die barrel, was also electrically heated but not cooled by air. The feed rate was 30 rpm. Extrusion temperature (ET) was defined as temperature at the die end of the barrel. Extrusion operation conditions were: ET, 125 °C and screw speed (SS) of 130 rpm. After extrusion the extrudates were cooled, equilibrated at environmental conditions (25 °C, RH = 65%), milled (UD Cyclone Sample Mill, UD Corp, Boulder, CO, USA) to pass through an 80-US mesh (0.180 mm) screen, packed in plastic bags, and stored at 4 °C.

### 3.3. Preparation of Amaranth Protein Hydrolysates

*In vitro* simulated digestion of amaranth protein was performed following the procedure by Megías *et al.* [36] with several modifications. Briefly, unprocessed and extruded amaranth flours were suspended in water (1:20 *w*/*v*) and a sequential enzyme digestion was carried out with pepsin (EC 3.4.23.1, 662 units/mg; enzyme/substrate, 1:20 (*w*/*w*); pH 2.0) and pancreatin (8× USP; enzyme/substrate, 1:20 (*w*/*w*); pH 7.5) at 37 °C for 3 h each. The hydrolysis with pepsin was stopped increasing the pH until 7.5 to start the hydrolysis with pancreatin, which was stopped by heating at 75 °C for 20 min, and the resulting hydrolysates were centrifuged at 20,000× *g* for 15 min at 4 °C. Aliquots of 50 mL were taken at 10, 25, 60, 90, 120 and 180 min during both hydrolysis, with pepsin and pancreatin. The total hydrolysis time was 360 min (180 min with pepsin followed by 180 min with pancreatin). Once the hydrolysates were desalted, they were freeze dried in a Labconco (Kansas, MO, USA) Freeze Dryer 4.5.

### 3.4. Peptide Characterization

For sample preparation, 1 mg of amaranth hydrolysates (UAH and EAH) were dissolved in 1 mL of deionized water and analyzed by using an UltrafleXtreme MALDI TOF/TOF mass spectrometer (Bruker Daltonics, Bremen, Germany) equipped with a frequency tripled Nd:YAG solid state laser using the FlexControl 1.4 software package (Bruker Daltonics). MS/MS analysis of each ion of interest, the peptide with the highest intensity under the curve, was performed at 500 Hz in LIFT mode using a randomized raster, summed and saved for analysis. Data processing was performed using the FlexAnalysis 3.4 software package (Bruker Daltonics, Bremen, Germany) and Biotools 3.2 (Bruker Daltonics, Bremen, Germany). The percentage of each peptide was calculated based on the area under the curve method. The total area for each sample was calculated by taking the sum of all the areas under the curve. Then the area under the curve for each peptide identified was divided by the total area and the percentage was calculated. Potential biological activities of the peptides were obtained using BIOPEP tool (http://www.uwm.edu.pl/biochemia/index.php/pl/biopep, accessed on 18 August 2014). Protein sequences were confirmed using BLAST^®^ tool (http://blast.ncbi.nlm.nih.gov/Blast.cgi).

## 4. Conclusions

The hydrolysis time impacted the peptide profile in both hydrolysates, from unprocessed and extruded flours. The extrusion process had a higher impact on the peptide profile, producing more peptides with lower MM and biological activity. The *in vitro* digestion with pepsin and pancreatin produced peptides with biological activity, which represented the simulated digestion and demonstrated that peptides could be formed during real digestion. In addition, the extrusion process was a good alternative as pre-treatment because it could break-down food proteins and made them more available to enzyme action. In summary, unprocessed and extruded amaranth flours are sources of peptides with potential biological activity, such as ACE-inhibitor and DPP-IV inhibitor, which are related with the prevention of important chronic diseases. Amaranth flours, unprocessed and extruded, as well as the peptides found in both flours, could be used as an ingredient or food supplement in a healthy diet to prevent the risk to develop chronic diseases.

## Abbreviations

ACE-Inhibitor: Angiotensin converting enzyme-inhibitor
DPP-IV-Inhibitor: Dipeptidyl peptidase IV inhibitor
EAH: Extruded amaranth hydrolysate
ET: Extrusion temperature
FAO: Food and agriculture organization
MALDI-TOF: Matrix-assisted laser desorption/ionization- time-of-flight mass spectrometer
MM: Molecular mass
MS-MS: Tandem mass spectrometry
RH: Relative humidity
rpm: Revolutions per minute
SS: Screw speed
UAH: Unprocessed amaranth hydrolysate
USP: Enzyme units (United States Pharmacopeia)
WHO: World health organization
